# Hospital mortality statistics in Tanzania: availability, accessibility, and quality 2006–2015

**DOI:** 10.1186/s12963-018-0175-3

**Published:** 2018-11-20

**Authors:** Irene R. Mremi, Susan F. Rumisha, Mercy G. Chiduo, Chacha D. Mangu, Denna M. Mkwashapi, Coleman Kishamawe, Emanuel P. Lyimo, Isolide S. Massawe, Lucas E. Matemba, Veneranda M. Bwana, Leonard E. G. Mboera

**Affiliations:** 1 National Institute for Medical Research, Headquarters, P.O. Box 9653, 11101 Dar es Salaam, Tanzania; 2 National Institute for Medical Research, Tanga Research Centre, P.O. Box 5004, Tanga, Tanzania; 3 National Institute for Medical Research, Mbeya Research Centre, P.O. Box 2410, Mbeya, Tanzania; 4 National Institute for Medical Research, Mwanza Research Centre, P.O. Box 1462, Mwanza, Tanzania; 5 National Institute for Medical Research, Amani Research Centre, P.O. Box 81, Muheza, Tanzania; 6 Southern African Centre for Infectious Disease Surveillance, Centre of Excellence for Infectious Diseases of Humans and Animals, P.O. Box 3297, Morogoro, Tanzania

**Keywords:** Hospital, Mortality, Cause of death, Data quality, Availability, Tanzania

## Abstract

**Background:**

Accurate and reliable hospital information on the pattern and causes of death is important to monitor and evaluate the effectiveness of health policies and programs. The objective of this study was to assess the availability, accessibility, and quality of hospital mortality data in Tanzania.

**Methods:**

This cross-sectional study involved selected hospitals of Tanzania and was carried out from July to October 2016. Review of hospital death registers and forms was carried out to cover a period of 10 years (2006–2015). Interviews with hospital staff were conducted to seek information as regards to tools used to record mortality data, staff involved in recording and availability of data storage and archiving facilities.

**Results:**

A total of 247,976 death records were reviewed. The death register was the most (92.3%) common source of mortality data. Other sources included the International Classification of Diseases (ICD) report forms, Inpatient registers, and hospital administrative reports. Death registers were available throughout the 10-year period while ICD-10 forms were available for the period of 2013–2015. In the years between 2006 and 2010 and 2011–2015, the use of death register increased from 82 to 94.9%. Three years after the introduction of ICD-10 procedure, the forms were available and used in 28% (11/39) hospitals. The level of acceptable data increased from 69% in 2006 to 97% in 2015. Inconsistency in the language used, use of non-standard nomenclature for causes of death, use of abbreviations, poorly and unreadable handwriting, and missing variables were common data quality challenges. About 6.3% (*n* = 15,719) of the records had no patient age, 3.5% (*n* = 8790) had no cause of death and ~ 1% had no sex indicated. The frequency of missing sex variable was most common among under-5 children. Data storage and archiving in most hospitals was generally poor. Registers and forms were stored in several different locations, making accessibility difficult.

**Conclusion:**

Overall, this study demonstrates gaps in hospital mortality data availability, accessibility, and quality, and highlights the need for capacity strengthening in data management and periodic record reviews. Policy guidelines on the data management including archiving are necessary to improve data.

## Background

Hospital management information systems is one key component of the Health Information System and is responsible for collection of data on vital registration of births and deaths occurring within the facility. Accurate and reliable information on the distribution, pattern, and causes of death is important to monitor and evaluate the health service performance and effectiveness of health policy and service delivery [1, 2]. Good quality hospital data is influenced by proper registration, storage, and archiving, as well as keeping storage areas clear and accessible. Proper record keeping and storage provide evidence of the hospital’s accountability for its actions; they form a key source of data for research, statistical reports, and health information systems [3]. It is important for saving time and resources. Moreover, sound and reliable information is essential for health system policy development and implementation, governance and regulation, health research, human resources development, health education and training, service delivery, and financing [2].

Globally, hospitals are among the largest producers of health data, generated daily by various levels of departments including outpatients, inpatients, and administrative units [4]. However, there are a number of challenges affecting hospital data generation, preservation, management, accessibility, and utilization [5–8]. The challenges include use of outdated registers/forms that require the need of constant revision; shortage of well-trained and experienced personnel; lack of planning in storage of inactive records; incompleteness of forms (missing of variables); inadequate storage facilities; and lack of determination of records retention period [6, 7, 9]. These weaknesses are compounded in continued use of paper-based hospital information systems in low-income countries that are prone to damage [5]. In most sub-Saharan countries, hospital related data are collected and reported using a paper-based subsystem for primary data collection.

Over the years, the Health Management Information Systems (HMIS) in low-income countries have remained paper-based which is cumbersome, are of uncertain reliability, and do not lend themselves to regular analysis or feedback to health care providers or planners [10]. Summarized data have been periodically submitted within an electronic subsystem, namely District Health Information System (DHIS2) for centralized aggregation of data, but only recently [11]. Studies in low- and middle-income countries have shown that HMIS systems are weak and characterized by incompleteness, poor quality, and limited utilization of data [12–19]. Moreover, in sub-Saharan Africa, preservation and conservation of hospital documents and records has posed a serious problem [8]. Due to poor archiving and preservation of hospital documents, accessibility and use of such data becomes a challenge.

Those in hospital management need timely and reliable information for planning and evaluating services. This is particularly important to achieve the Sustainable Development Goals (SDGs), which require timely and accurate data in order to facilitate evidence-based policymaking [20]. Despite this, in sub-Saharan Africa, routine health data of acceptable quality are usually unavailable and underutilized. Data collected through the routine HMIS are rarely complete and usually not representative. Fundamental changes in health care delivery have resulted in a critical need for evidence-based decision-making from reliable data and information. However, the quality of such decisions depends upon the quality of the data generated by the information systems. Despite the usefulness of hospital mortality data in monitoring and evaluation of health care services, there is a dearth of information on the availability, accessibility, and quality of hospital mortality data in most sub-Saharan Africa. The objective of this study was to assess the availability, accessibility, and quality of hospital mortality data in Tanzania for a period of 2006–2015.

## Methods

### Study sites

**Fig. 1:**
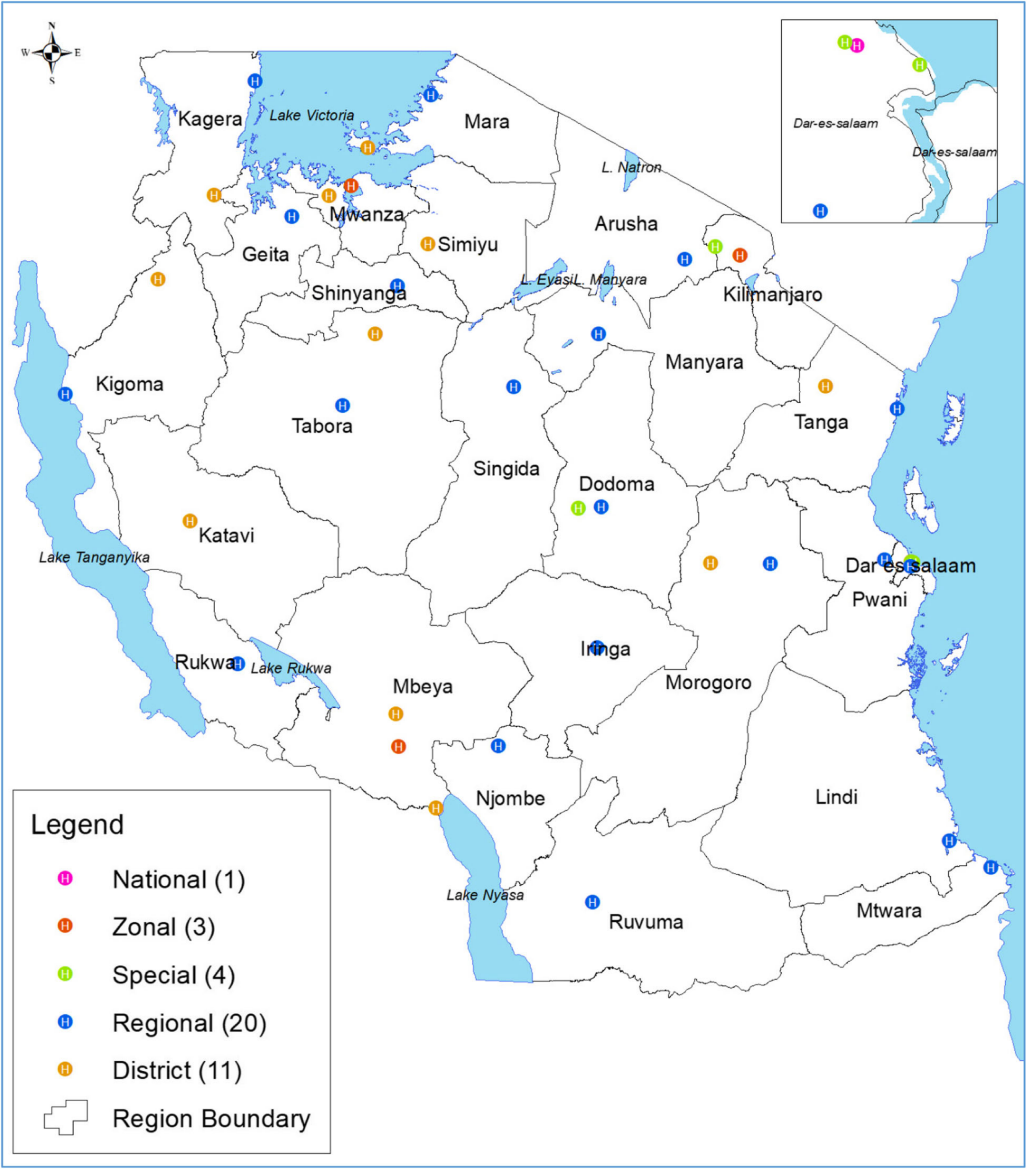
Map of Tanzania showing the hospitals in the study

This retrospective study was carried out from July to December 2016 and involved 39 public hospitals of different levels (Fig. 1). These were one national hospital (Muhimbili), three zonal referral (Bugando Medical Centre, Mbeya Referral Hospital, and Kilimanjaro Christian Medical Centre), four special hospitals (Muhimbili Orthopaedic Institute, Ocean Road Cancer Institute, Mirembe Mental Hospital, and Kibong’oto Infectious Disease Hospital), 20 regional referral hospitals (Temeke, Kagera, Kitete (Tabora), Morogoro, Maweni (Kigoma), Dodoma, Bombo-Tanga, Mara, Mount Meru-Arusha, Shinyanga, Manyara, Ruvuma, Singida, Geita, Ligula (Mtwara), Tumbi (Pwani), Rukwa, Iringa, Sokoine (Lindi), and Njombe) and 11 district hospitals (Sengerema, Ukerewe, Mpanda, Kyela, Chunya, Biharamulo, Nzega, Kilosa, Kibondo, Lushoto, and Maswa). There are 269 hospitals in Tanzania, of which 120 (44.6%) are public and the remaining 149 (55.4%) are privately owned either by faith-based organizations, private*-*for-profit, or non-governmental organizations.

National, zonal referral, and special hospitals were included conveniently. To select sites from regions and districts, a multistage sampling technique with a set of guiding inclusion criteria was employed. All regions were included to ensure that geographical representation is captured. Epidemiological profile of diseases with high morbidity and main causes of deaths, ecological factors, the population of the area, human resources coverage, and other spatial variations were also considered to decide on the number of hospitals to be included in each region to show a national representative sample. In regions or districts where zonal referral hospitals are located, the respective region/district hospitals were not included.

### Data collection procedure

#### Extraction processes

Data were collected using customized paper-based collection tools. The research team and data collectors were trained on use of data collection tools, how to review hospital registers and reporting forms, the types of data required, the data extraction process, and agreed-upon documentation of data quality issues. Data was collected for patient’s age, sex, name and level of hospital, and cause and date of death. Sources of data included death registers, inpatient registers, International Classification of Diseases (ICD-10) report forms, patient files, and/or administration reports. A thorough search, guided by a hospital staff, of the tools (registers and forms) used to record mortality data was conducted in all hospitals. This involved going through all mentioned storage facilities to collect and compile all found forms and registers into a single database. The extraction process started with the source with the largest number of records based on discussion with the key members of the hospital management team and review of what had been compiled. A monthly checklist was done to mark data completeness status (by date) for that source. The next source was then taken to fill time periods (dates) where no data were found from the previous one. This iterative process was done until all identified sources were fully assessed and reviewed.

#### Interviews and observations

A guided questionnaire was used to conduct interviews with the hospital staff. Information collected during the interviews included patterns of deaths (average per week/month, age group mostly affected, and wards with large number of events), tools (registers and forms) used to record mortality and causes of death data, issues associated with tools availability, procedure and time of filling the information, staff involved in recording, quality of record keeping, availability of data storage, and availability of archiving facilities. All the registers and report forms were reviewed for recording patterns, completeness, storage and archiving, period covered, and data volume.

### Data management

Data were entered using a database developed in EpiData software (version 3.1, EpiData Association, Odense, Denmark). Data were checked for immediate errors before entry. Data clerks were oriented before starting the work. Data were distributed to clerks in a specific order to facilitate a quality check exercise. A quality check was done by taking a proportion of entered data and compared with original data. This was done for 1%, then increased to 2%, then up to 3% where it was found necessary. After entry, all data were migrated to STATA version 13 (Stata Corporation College Station, TX, USA) for further processing and analysis.

Descriptive analysis was done to document patterns in data accessibility, availability, and quality. Accessibility was measured using field documentation on time taken to get the required data at the hospital, the complexity and difficulties encountered in searching for the registers, and nature and places used for storage and archiving. To assess availability, an assumption was made that at least a single death (of any age) would occur in a hospital within a month; therefore, for each year, a minimum of 12 death events were expected. For each hospital, a year with less than 12 death events was marked as *“not acceptable*,” and a year where no documentation on death events was done was named as *“no data.”* Data quality was assessed by summarizing entries which did not include sex, age, date, or cause of death. For some comparison purposes, the study period was categorized into two five-year windows (2006–2010 and 2011–2015).

## Results

### Data availability

A total of 247,976 death records were reviewed. The death register was the most (92.3%) common source of mortality data. Other sources were in-patient registers, ICD-10 report forms, and administrative and supervision reports. Table 1 depicts comparison between ICD-10 report forms and death registers for different attributes.

**Table 1:** Characteristics and status of ICD-10 report form and death registers, 2006–2015

Characteristic	ICD-10 report form	Death registers
Usage (2006–2010)	Introduced in November 2012	Used by 82% (32/39) of all hospitals
Usage (2011–2015)	Used by 28% (11/39) of all hospitals	Used by 94.9% (37/39) of all hospitals
Completion time	Completed at clinician’s convenient time	Completed as soon as the death event occurs
Staff responsible for recording	Completed by nurses, recorders, or HMIS staff	Completed by clinician on call
Storage and archiving	Stored in hospital wards	Stored either in hospital wards, Hospital Registry (Medical Records) Unit, or Office of District Commissioner
Data volume recorded	Low data compared to those available in death registers	More complete data (large number of cases)
Form format	Different versions found in use with different variables	Standardized format and variables
Stock-out	Stock-out commonly reported	Readily available

There was improvement in recording cases of death using the death register over time. During the period of 2011–2015, the use of ICD-10 report forms was still minimal (28%) while almost all hospitals (94.9%) were using death registers. Interestingly, 3 years after the introduction of ICD-10 report forms, different versions of ICD-10 report forms were used. While the death registers were reported to be completed immediately after death occurrence, the ICD-10 report forms were completed at any convenient time after death. The delay in completion of ICD-10 report forms was described to be due to lack of urgency or lack of immediate use of data collected (Table 1).

Eight hospitals including one zonal referral hospital, one special hospital, three regional referral hospitals, and three district hospitals presented low availability of data from death registers. Chunya and Tumbi had an extreme situation where data for 10 and 9 years, respectively, could not be located (Table 2). Among reported reasons for low level of availability were that data were either destroyed (hospitals = 2), records moved to a place outside the hospital (1), poor storage capacity (6), and that data could not be found (4).

**Table 2:** Hospitals with lowest availability levels from death registers

Name of hospital	Level of hospital	No. years with no data	Period
Chunya	District	10	2006–2015
Tumbi	Regional	9	2006–2014
Kibondo	District	6	2006–2011
KCMC^a^	Zonal	5	2006–2010
Mount Meru	Regional	5	2006–2010
Njombe	Regional	5	2006–2010
Lushoto	District	5	2006–2007, 2010–2011, 2015
MOI^b^	Special	5	2006–2010

^a^*KCMC* Kilimanjaro Christian Medical Centre

^b^*MOI* Muhimbili Orthopaedic Institute

Hospitals with the highest availability ICD-10 report forms included only three regional referral hospitals and one district hospital (Table 3). Some hospitals were found to use ICD-10 report forms for only 1 year or just few months.

**Table 3:** Hospitals with highest availability levels of ICD-10 report form

Name of hospital	Level of hospital	No. of years with data	Period
Manyara	Regional	3	2013–2015
Maswa	District	3	2013–2015
Morogoro	Regional	3	2013–2015
Sumbawanga	Regional	3	2013–2015
Kyela	District	2	2014–2015
Mpanda	District	2	2014–2015
Sengerema	District	2	2014–2015

**Fig. 2:**
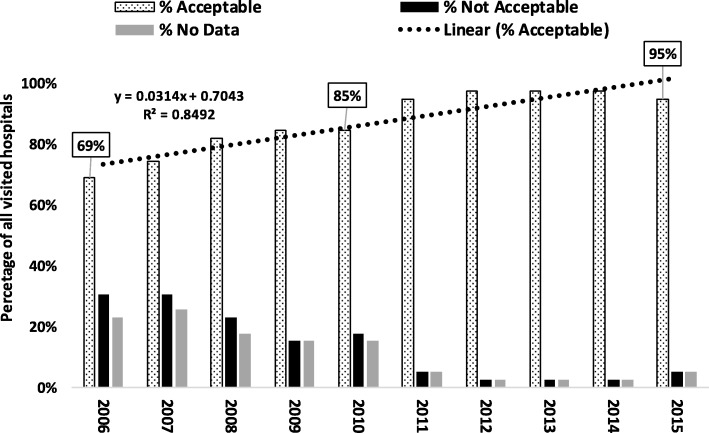
Pattern of hospital mortality data availability in Tanzania, 2006–2015

Results on data acceptability and available assessment are presented in Fig. 2. The level of data acceptability increased from 69% in 2006 to 85% in 2010 (a 16.8% increase in a period of 5 years) and to 95% in 2015 (a 27.4% increase in a period of 10 years). Hospital mortality data for 2006 and 2007 were the most difficult to access (high proportion of hospitals with “*not acceptable*” and “*no data*”). However, that decreased over time, and dropped drastically after 2010 (Fig. 2), indicating improvement in either recording or keeping.

### Data quality

**Fig. 3:**
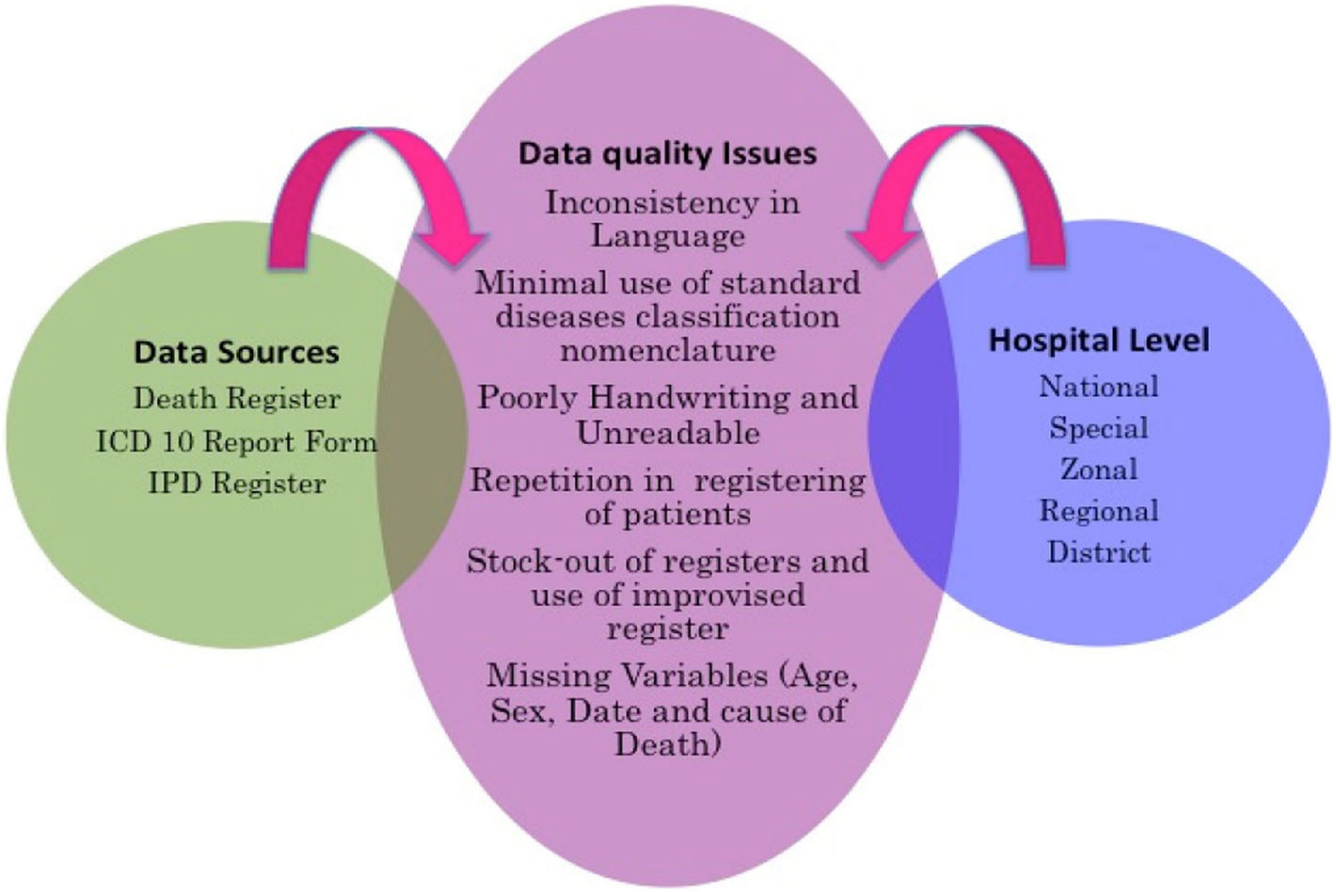
Quality challenges in hospital mortality data

Quality of data was a challenge in all used sources of data (death register, ICD-10 report form, and IPD register) and in all hospital levels (Fig. 3). Inconsistency in the language used to fill mortality data was found where either English or Kiswahili or both were used. Use of non-standard nomenclature for writing diagnoses was common in almost all hospital levels. This was observed in 12 of the 20 regional referral hospitals (Manyara, Singida, Tabora, Shinyanga, Kigoma, Iringa, Arusha, Rukwa, Mtwara, Lindi, Ruvuma, and Temeke). Difficult to read handwriting and use of abbreviations were also a challenge in recording these data. Use of abbreviations was common at Muhimbili National Hospital and Kilimanjaro Christian Medical Centre (KCMC). Repetition in registering of patients was common, resulting in the same patient being recorded more than once. This was mostly observed in zonal referral hospitals.

Stock-outs of registers and report forms were reported to be common in four regional referral hospitals (Morogoro, Dodoma, Rukwa, and Iringa). When improvised registers were used, there was inconsistency in the format used, resulting in missing some of the variables and important information.

**Fig. 4:**
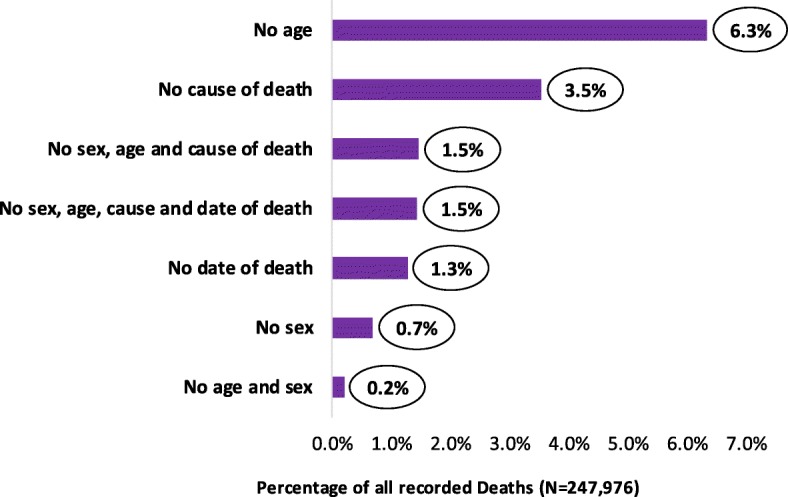
Percentage of records with missing variables in collected hospital mortality data

**Fig. 5:**
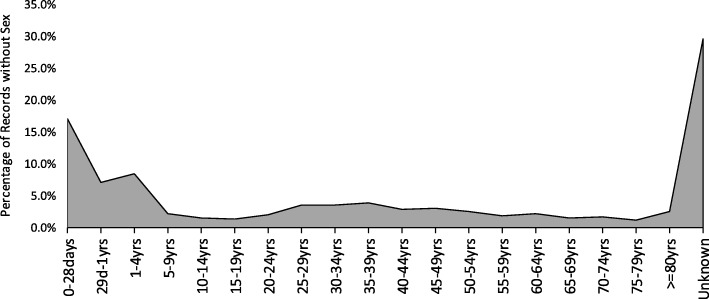
Pattern of records without sex by different categories of age

Missing variables included patient age, sex, date of death, and cause of death (Fig. 4). The most missed variable was the age of the patient (6.3%, *n* = 15,719) followed by deaths with no cause specified (3.5%, *n* = 8790). Over 3500 cases (> 1.5%) were recorded without sex, age, cause of death, and date of death (Fig. 4). Examining the pattern of missing sex variable, the findings indicate that the frequency was most common among children under 5 years and those whose age were not recorded (Fig. 5). A total of 224,253 records (90.4%) had all the four core variables.

### Data accessibility, storage, and archiving

**Fig. 6:**
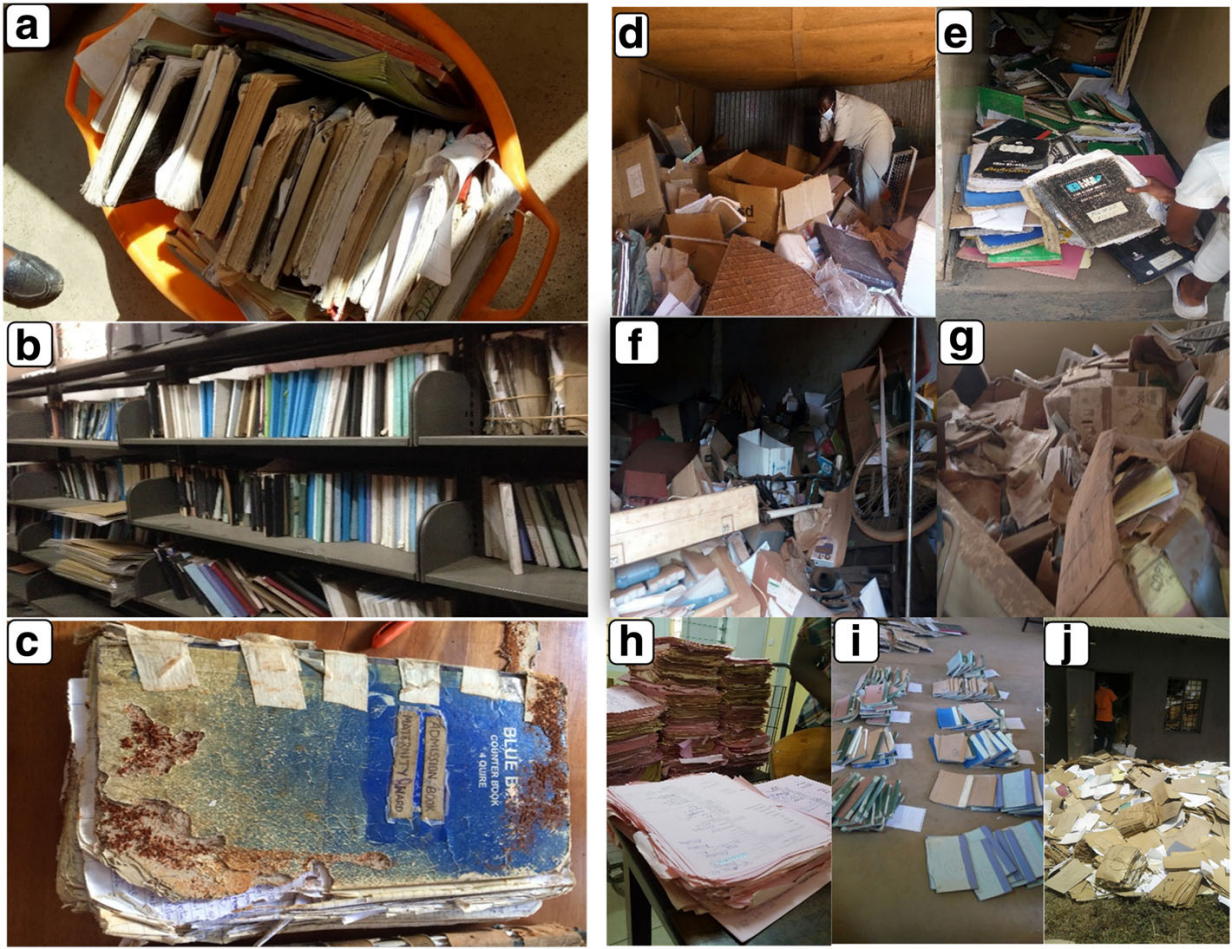
Storage of registers and forms in some of the hospitals in Tanzania. **a** Different types of registers compiled from different places kept in a plastic container; **b** One stop point designed for data storage; **c** Inpatient register that was destructed by ants; **d**, **e** Searching for death registers in a hospital store; **f**, **g** Storage of used registers with disposed equipment; **h**, **i**, **j** Sorting of death report forms

Generally, data accessibility was a big problem in some hospitals and varied between hospitals and levels of hospitals. There were unclear allocation of designated premises for data storage. Registers were stored in different location; some were found in medical record units (MRU), the mortuary, or in the wards. Hence, several places had to be searched to collate the needed data. Data that were kept in hospital wards could be easily collected; however, in most sites, these were those of recent months or hardly two to 3 years old. In most cases used registers and those of past 6–10 years were kept outside the wards. This was mentioned to be due to lack of space for filling all data (Fig. 6). In special hospitals, i.e., ORCI, MOI, Mirembe, and Kibong’oto, the search was not too hectic which could be due to less patients and more wards available. For instance, in Mirembe and Kibong’oto, registers were kept in the MRU and were readily available and properly stored. In over 55% (11/20) of regional referral hospitals, registers were kept in the hospital wards. Of the remaining regional referral hospitals, registers were recorded in the MRUs in three hospitals, and at another, registers were recorded in both the hospital wards and MRUs. In all district hospitals, registers were mainly kept in wards. In three hospitals (Tumbi, Kigoma, and KCMC), some of the death registers were kept in the mortuary unit.

In other hospitals, sources of mortality data were found to be lumped together in one of the old rooms at the hospital or areas where all other unwanted stuff such as old equipment, bicycles, and beds were stored (Fig. 6). This resulted in more time being allocated for searching as well as through sorting through of materials to get the needed data. This process happened at hospitals in Shinyanga, Njombe, Lindi, and Nzega. In some hospitals, it took two to 3 days to complete the search. Among the three zonal referral hospitals, records storage and archiving at Bugando Medical Centre was observed to be very good. Despite the size of the data available, it took half a day to obtain all needed information. At KCMC and Mbeya Zonal Referral hospital, it took over two days to search for the data; even then, there remained a lot of missing data. Kagera and Singida hospitals were also found to have very good data management.

Data storage and archiving in regional referral hospitals was generally poor. In eight (8/20) hospitals (Tumbi, Morogoro, Dodoma, Njombe, Iringa, Mara, Geita, and Temeke), there was improper storage of the registers. Many registers were lost or misplaced, even for the most recent years. Some hospital management teams explained that the missing data was due to construction or renovation of hospital buildings, resulting in reallocations of ward and offices and losing records in the process. In Manyara, Kigoma, Tabora, Rukwa, Arusha, Ruvuma, and Mtwara, registers were stored in the respective wards. Shinyanga and Temeke hospitals did not have an archival space for registers and other documents. Of all regional referral hospitals, Kagera had a well-constructed storage facility. Lack of room for archiving medical records was frequently cited by most of the hospital staff as a factor contributing to loss of old patient and other important hospital records. Of the 11 district hospitals, five (Nzega, Kibondo, Kyela, Biharamulo, and Lushoto) did not have storage or archiving facilities for medical record files and registers. The documents were lumped together in a small room where all other documents and equipment were stored.

In two district hospitals (Kilosa and Maswa), stored medical records, including registers, were in very poor condition and infested with white ants. Most of the registers in Chunya district hospital were not available and reported to have been destroyed by burning to allow hospital building renovation. Knowledge of the hospital staff on data storage and improper hand-over procedures were also some of the challenges in data storages and hence accessibility. Hospitals lacked clear archiving guidelines, especially as the allowable duration to keep the documents caused some records to be destroyed or moved to a place where they were prone to destruction and could not be easily traced.

## Discussion

At almost every hospital, data sources could not be readily accessed, were poorly organized, and contained incomplete data. Data availability varied between hospitals and levels of hospitals. Although health information systems are widely considered to be a foundation of public health with available, reliable, timely, and valid data [21], several studies indicate that low quality of data is common among many low- and middle-income countries [22–24]. Incompleteness has been identified as the most common causes of poor data quality in resource-poor countries [25–27]. In the current study, incompleteness was observed in almost all of the 39 hospitals surveyed. This observation was also noted even within the hospitals that collect and store data in the electronic-based District Health Information System (DHIS2). Similar observations have been reported elsewhere in sub-Saharan Africa [28].

The findings of this study have indicated that in almost all hospitals, there was minimal use of standard disease classification nomenclature and with inconsistency in the language used. Tanzania introduced the use of International Classification of Diseases (ICD) version 10 in November 2012, yet two thirds of the hospitals were not using the system during the time of this study. The ICD-10 aims to promote international comparability in the collection, processing, classification, and presentation of mortality statistics [29]. It is a requirement by the World Health Organization that the underlying cause of death (UCoD) are coded according to the ICD procedures [30]. Studies elsewhere have reported that the rules of UCoD determination for several causes of death are not consistent across different death registration systems, making comparisons difficult [31, 32]. In an analysis of mortality data for 69 countries, Lu et al. [32] found that many countries fail to report sufficiently specific codes in mortality data. The minimal use of ICD-10 observed in our study has been reported elsewhere in low- and middle-income countries. Studies in Sri Lanka [33], Thailand [34], and China [35] have revealed massive misclassification of the cause of death in hospitals. On the contrary, a recent study in Nigeria, reported a high level of nationwide implementation of ICD-10 [36].

Generally, poor quality of data was observed in the majority of hospitals in Tanzania. The poor quality of hospital data has been attributed to the fact that custodians of mortality data systems do not realize the importance of periodically assessing the accuracy of hospital cause of death data, and that health care providers in hospitals may lack the time, incentives, diagnostic facilities, or training to correctly certify causes of death, in addition to rarely understanding that their diagnoses guide national health priorities [33]. Most often, medical records’ departments, which code death certificates and compile the data into cause of death, are often understaffed, lack rigorous statistical protocols for checking data quality, and may not appreciate the epidemiological and statistical importance of their work. In a study in Nigeria, it was found that although the information contained in medical record is considered to be essential, but the process of documenting it is often considered a lesser priority by many health caregivers [37]. Studies elsewhere have described several factors affecting data quality to include little attention paid to this issue even in policy [11, 38], infrequent supervisory support that might correct faults and weaknesses in the health information system.

In this study, in some district hospitals, death certification was only done upon requests from the family and relatives of the deceased. Although more than three quarters of deaths are registered in most African countries [39], only 2% of the countries have complete death registration data and half of the countries record no cause of death data [40]. Similar to the findings of this study, in Malaysia, some of the factors contributing to poor quality of data included: listing a mode of death rather than a cause of death, incorrect sequencing of causes of death, despite the mention of the actual underlying cause of death, and inconsistency with underlying cause of death [34]. Other errors frequently observed in our study were the use of abbreviations, ambiguous terms, and poor handwriting. Most often, in clinical records, many items are handwritten and difficult to read. All these contribute to the challenges in the accurate identification of underlying causes of death from original death registers. With poor handwriting, much information in medical records is inaccessible to other clinicians, medical auditors, or researchers. Similar observations have been reported elsewhere [41–45].

Findings of this study indicate that data accessibility was poor. In data quality context, data accessibility is defined as the range to which data are available or easily and quickly retrievable [46]. Poor accessibility of hospital mortality data was attributed to the poor storage and archiving facilities. This has affected the accessibility and availability of mortality data in this study. In some of the hospitals, registers, reporting forms, and other records were not properly taken care of, misplaced, or thrown away when buildings were undergoing renovations. It is a known fact that, hospitals are amassing data at an unprecedented rate, but most of them do not have governance in place to ensure that data is recorded, used, stored, and archived appropriately. Despite the fact that keeping good quality hospital data is an essential component of hospital management, in low-and middle-income countries, it is often neglected [6, 8, 47–49]. We have identified main challenges in preservation and management of hospital data in low-income countries, including: use of outdated forms, inadequate human resources, lack of planning in storage of inactive registers and report forms, and lack of determination of record and register retention period. Poor preservation and archiving of hospital registers and records is also likely to be attributed to lack of legislation and policy guidelines on the data retention period or poor enforcement of the regulations [48]. One of the biggest challenges in preservation and archiving of hospital records in developing countries is educating the record keepers on the best ways to handle hospital records. This challenge is exacerbated by the fact that preservation of records is not at the centre of most medical science curricula. Formal specialized training on preservation and archiving of records are rare in many sub-Saharan African countries [50].

The findings of this study have highlighted the challenges in recording, quality, storage and archiving of hospital mortality data in Tanzania. Recognizing these challenges presents an important opportunity toward improving hospital data management and use, from the planning of enquiry and reporting systems through data collection, processing, and compilation, to making data available in the public domain for wider use. Successful record-keeping requires the service of the hospital management team and health providers who can appropriately organize information in hospitals. Mortality measurement will further gain through creating opportunities for expanding hospital management team knowledge about the usefulness of reliable mortality data [51]. Through education and training, the clinicians, nurses, and information managers will be able to acquire skills to improve the present situation of information management in the hospitals. It is important, therefore, that the knowledge and skill of hospital record management and preservation is incorporated into the hospital staff through formulation of data management policies, training, infrastructure development, and provision of an adequate budget. Strategic strengthening of analytical capacity at the hospitals and national levels should be emphasized. It is equally important that registration of causes for death occurring in hospitals require periodic validation prior to their use for public health policy [51]. Training and instilling a culture of valuing data are will help to solve the problem of poor hospital record preservation and management, as well as its staff negative impact on health care delivery. The adoption of electronic information systems is likely to revolutionize efforts to strengthen the Health Management Information Systems [52]. There are currently major efforts to digitize hospital records in Tanzania, but without improvement in the quality of paper records, the full benefits of digitization are unlikely to be achieved.

## Conclusion

In conclusion, this study illustrates that causes of death are not accurately reported in hospitals of Tanzania, mainly due to non-adherence to the International Classification of Diseases and poor data management. To improve the reliability and usefulness of hospital mortality data, the government should strengthen the capacity of health workers in data management and make available standard registers and forms. It is important to also strengthen supportive supervision and use periodic hospital record reviews to validate the quality of the data.

## Abbreviations

DHIS: District Health Information System
HMIS: Health management information systems
ICD: International Classification of Diseases
KCMC: Kilimanjaro Christian Medical Centre
MOI: Muhimbili Orthopaedics Institute
MRU: Medical Records Unit
ORCI: Ocean Road Cancer Institute
SDG: Sustainable Development Goals
UCoD: Underlying cause of death
